# Bubble CPAP to support preterm infants in rural Rwanda: a retrospective cohort study

**DOI:** 10.1186/s12887-015-0449-x

**Published:** 2015-09-24

**Authors:** Evrard Nahimana, Masudi Ngendahayo, Hema Magge, Jackline Odhiambo, Cheryl L. Amoroso, Ernest Muhirwa, Jean Nepo Uwilingiyemungu, Fulgence Nkikabahizi, Regis Habimana, Bethany L. Hedt-Gauthier

**Affiliations:** Partners In Health/Inshuti Mu Buzima, Rwinkwavu, Rwanda; Ministry of Health, Kigali, Rwanda; Division of General Pediatrics, Boston Children’s Hospital, Boston, USA; Division of Global Health Equity, Brigham and Women’s Hospital, Boston, USA; Department of Global Health and Social Medicine, Harvard Medical School, 02115 Boston, MA USA

**Keywords:** bCPAP, Very low birth weight, Preterm, Premature, Respiratory distress, CPAP, Rwanda, Africa

## Abstract

**Background:**

Complications from premature birth contribute to 35 % of neonatal deaths globally; therefore, efforts to improve clinical outcomes of preterm (PT) infants are imperative. Bubble continuous positive airway pressure (bCPAP) is a low-cost, effective way to improve the respiratory status of preterm and very low birth weight (VLBW) infants. However, bCPAP remains largely inaccessible in resource-limited settings, and information on the scale-up of this technology in rural health facilities is limited. This paper describes health providers’ adherence to bCPAP protocols for PT/VLBW infants and clinical outcomes in rural Rwanda.

**Methods:**

This retrospective chart review included all newborns admitted to neonatal units in three rural hospitals in Rwanda between February 1st and October 31st, 2013. Analysis was restricted to PT/VLBW infants. bCPAP eligibility, identification of bCPAP eligibility and complications were assessed. Final outcome was assessed overall and by bCPAP initiation status.

**Results:**

There were 136 PT/VLBW infants. For the 135 whose bCPAP eligibility could be determined, 83 (61.5 %) were bCPAP-eligible. Of bCPAP-eligible infants, 49 (59.0 %) were correctly identified by health providers and 43 (51.8 %) were correctly initiated on bCPAP. For the 52 infants who were not bCPAP*-*eligible, 45 (86.5 %) were correctly identified as not bCPAP-eligible, and 46 (88.5 %) did not receive bCPAP. Overall, 90 (66.2 %) infants survived to discharge, 35 (25.7 %) died, 3 (2.2 %) were referred for tertiary care and 8 (5.9 %) had unknown outcomes. Among the bCPAP eligible infants, the survival rates were 41.8 % (18 of 43) for those in whom the procedure was initiated and 56.5 % (13 of 23) for those in whom it was not initiated. No complications of bCPAP were reported.

**Conclusion:**

While the use of bCPAP in this rural setting appears feasible, correct identification of eligible newborns was a challenge. Mentorship and refresher trainings may improve guideline adherence, particularly given high rates of staff turnover. Future research should explore implementation challenges and assess the impact of bCPAP on long-term outcomes.

## Background

Over 2.9 million neonatal deaths occur every year, representing 44 % of all under five deaths [1–3]. In Rwanda, despite a rapid decline in under-five mortality, the number of deaths in the neonatal period remains high (27/1000 live births) with little change over the past 10 years [4, 5]. Major causes of neonatal deaths include preterm birth, birth asphyxia and infections. Recently, complications related to prematurity have surpassed pneumonia and diarrheal diseases as the number one cause death in children, and account for 35 % of all neonatal deaths [1–3, 6–8]. Hospital-based interventions targeting these causes are needed to reduce neonatal mortality, particularly in low and middle income countries [9–11].

The implementation of hospital-based interventions is challenging in resource limited settings. Specifically, intensive care unit technology for respiratory distress, such as a mechanical ventilation, is often not available due to high costs, maintenance demands and the need for highly trained staff. However, continuous positive airway pressure (CPAP) has been demonstrated to be a simple, low-cost and effective alternative to improve the respiratory status of preterm infants with respiratory distress syndrome [12, 13], and decrease the need for conventional mechanical ventilators [12, 14]. CPAP helps keep the respiratory tract and lungs open, promotes comfortable breathing, improves oxygen levels and decreases apnea in premature infants. Bubble CPAP (bCPAP) is the least expensive and least complicated CPAP option, making this the preferred technology in resource-limited settings [15, 16].

To date, few studies have been conducted to show the impact and feasibility of bCPAP in areas with limited resources. These studies, most of which were conducted in teaching and/or urban hospitals, have shown that bCPAP can reduce the need for mechanical ventilation and can be applied by nurses after a short on-the-job training on the protocol and equipment [12, 17]. However, little research has been done on the use of bCPAP in rural resource-limited settings and hospitals without pediatric specialists.

In January 2013, the Rwandan Ministry of Health (MOH), in collaboration with Partners In Health (PIH), introduced a bCPAP program integrated into broader neonatal care services for newborns with respiratory distress in three rural district hospitals (Butaro, Kirehe and Rwinkwavu District Hospitals). Nurses and general practitioners working in the neonatal units in these hospitals with a background in neonatal care services received intensive training on advanced neonatal care, focusing on the bCPAP protocol, safe assembly, maintenance and trouble-shooting of different issues related to bCPAP use. The training was supplemented by ongoing clinical mentorship and intermittent refresher trainings led by PIH and local MOH bCPAP champions.

The objectives of this study are to describe the provider adherence to bCPAP protocol for preterm and very low birth weight (PT/VLBW) infants and to describe the outcomes of these infants at the three district hospitals. The ultimate goal is to better understand the use of bCPAP in rural resource-limited settings in order to improve the quality of bCPAP implementation and inform the scale-up of this technology in similar settings.

## Methods

This retrospective cohort study included infants receiving care at neonatal units at Rwinkwavu, Kirehe and Butaro District Hospitals from February 1, 2013 to October 31, 2013. The catchment area included 865,000 people and care at the hospital was obtained after referral from one of the 41 health centers within the districts. These three hospitals were selected for the study as they were the only rural district hospitals providing basic neonatal care using bCPAP in Rwanda in 2013. A team of nurses and general practitioners worked permanently in these units providing care to an average of 25 infants every month in each hospital. Infants who needed intensive neonatal care, including mechanical ventilators, were referred to tertiary hospitals in Kigali city (the capital of Rwanda). Following the training on implementation of bCPAP, Rwinkwavu and Kirehe District Hospitals benefited from fairly consistent mentorship from PIH pediatric specialists during the study period while Butaro hospital had more intermittent specialist presence.

**Fig. 1:**
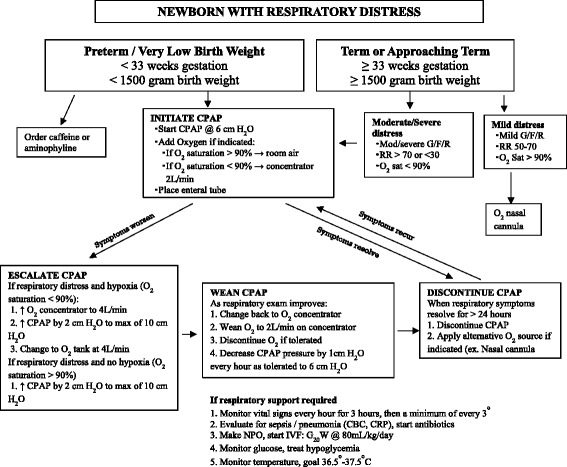
CPAP indication and implementation for newborns with respiratory distress based on the Rwanda CPAP protocol 2013

Respiratory assessment to determine the need for bCPAP is based on physical examination (such as grunting, nasal flaring and chest retraction) and vital signs (including respiratory rate and/or oxygen saturation). In addition, the etiology of respiratory symptoms and the natural history of that diagnosis are considered. Once the overall assessment is complete, the degree of respiratory distress is categorized as mild, moderate or severe. Moderate to severe signs include moderate to severe grunting, flaring, retractions and respiratory rate >70 or <30 and/or oxygen saturation <90 % (The oxygen saturation was measured using pulse oximeter). Based on the bCPAP protocol used in the three district hospitals, any newborn with a moderate to severe respiratory distress should have been initiated on bCPAP (Fig. 1). Furthermore, preterm (gestational age (GA) <33 weeks) or very low birth weight (<1500 g) infants with any degree of respiratory distress (mild, moderate or severe) should have been initiated on bCPAP. Preterm infants with significant apnea and bradycardia of prematurity were also eligible.

Our study population included all PT/VLBW infants admitted in neonatology units at the three hospitals. All term and near term infants (GA ≥33 weeks and/or birth weight ≥1500 g) were excluded as the severity of their respiratory distress was not captured in the patient charts and therefore eligibility for bCPAP could not be ascertained. For infants included in the study, we added a category of unknown to indicate missing data. The following information was extracted from the patient charts and registers in the neonatology and maternity unit: place of birth, birth weight, gestational age, respiratory rate, oxygen saturation, presence of physical signs of respiratory distress (grunting, chest retraction, nasal flaring), bCPAP recommendation and initiation, final disposition (recovered, referred or died) and presence of bCPAP complications (skin injury, pneumothorax, abdominal distention). We categorized PT/VLBW infants with at least one sign of respiratory distress as *bCPAP eligible* and those without any sign of respiratory distress as *bCPAP ineligible*. Data was extracted into a standard data collection form, and a file linking the study ID to the mother and neonate ID was kept separately during the data collection and destroyed after data validation. We analyzed data using Stata 12.1 (College Station, TX: StataCorp LP). We used descriptive statistics reporting number and percent of infant characteristics, infants identified as eligible for bCPAP, infants for whom bCPAP was initiated and clinical outcomes based on CPAP eligibility. We also used median and interquartile range for the duration of stay in the hospital.

The study received technical and ethical approvals from Rwanda institutional review boards: The Inshuti Mu Buzima Research Committee (IMBRC), the National Health Research Committee (NHRC) and the Rwanda National Ethics Committee (RNEC). As the study used de-identified routinely corrected data, the consent for parents was waived. STROBE (STrengthening the Reporting of OBservational studies in Epidemiology) guidelines were also followed for this study.

## Results

During the study period, 862 infants were admitted in the three hospitals. Of these, 136 (16 %) were identified as PT/VLBW and included in the analysis (Table 1). Of the 136 infants, 75.7 % (*n* = 103) were VLBW and 57.4 % (*n* = 78) were preterm. Most of the PT/VLBW infants (*n* = 117, 86 %) were born at a health facility, either hospital or health center. The median number of days of stay at the hospital was 19 with an interquartile range of 6–32 days. In assessing the presence of respiratory distress symptoms among PT/VLBW infants, 61.0 % (*n* = 83) showed at least one sign of respiratory distress (Table 2). Many of the infants (50.7 %, *n* = 69) had low oxygen saturation (SpO_2_ <90 %) and 38 infants (28.4 %) had chest retraction. In some cases, the clinicians only mentioned infants in respiratory distress without specifying the physical symptoms. One infant did not have documentation of the presence or absence of respiratory distress and thus, bCPAP eligibility could not be determined.

**Table 1:** Characteristics of infants admitted to the neonatology unit in three district hospitals in Rwanda

Population characteristics	Preterm or very low birth weight infants		Term and near term infants who are not very low birth weight	
	*N* = 136		*N* = 726	
	n	%	n	%
Place of birth				
Hospital	66	48.5	419	57.7
Health center	51	37.5	255	35.1
Home	14	10.3	32	4.4
Unknown	5	3.7	20	2.8
Birth weight				
Very low birth weight (<1500 g)	103	75.7		
Low birth weight (1500–2499 g)	31	22.8	224	30.9
Normal birth weight (>=2500 g)	2	1.5	437	60.1
Unknown	0	0	65	9.0
Gestation age at birth				
<33 weeks	78	57.4		
33–36 weeks	19	14.0	71	9.8
=37 weeks	9	6.6	548	75.5
Unknown	30	22.1	107	14.7
Duration of stay in the hospital	*N* = 128		*N* = 705	
Median (IQR)	19 (6–32)		7 (3–10)	
0–7 days	34	25.0	413	56.9
8–14 days	18	13.2	140	19.3
15–30 days	34	25.0	52	7.2
>30 days	29	21.3	33	4.5
Unknown	21	15.4	88	12.1

**Table 2:** Evidence of respiratory distress among preterm (<33 weeks) or very low birth weight (<1500 g) infants

Sign of respiratory distress (*N* = 136)	Infants with symptoms	
	n	%
SpO_2_ <90 %	69	50.7
Grunting (*N* = 134)	17	12.7
Chest retraction (*N* = 134)	38	28.4
Nasal flaring (*N* = 134)	15	11.2
Respiration rate <30 or >70	24	17.7
At least one sign of respiratory distress (*N* = 135)	83	61.5

Of the 135 PT/VLBW infants whose bCPAP eligibility could be determined, 61.5 % (*n* = 83) were bCPAP-eligible of which 59.0 % (*n* = 49) were correctly identified by health providers and for 51.8 % (*n* = 43) bCPAP was initiated. Twenty-three bCPAP-eligible infants (27.7 %) had no indication of being identified as bCPAP eligible or of being initiated on bCPAP. Information around identification was missing for 13.3 % (*n* = 11) of infants who were eligible (Table 3). For the 52 infants who were not bCPAP*-*eligible, 45 (86.5 %) were correctly identified as not bCPAP-eligible and 46 (88.5 %) did not receive bCPAP.

**Table 3:** bCPAP identification and initiation for preterm (<33 weeks) or very low birth weight (<1500) infants

	bCPAP eligible		bCPAP Not eligible		Total	
	*N* = 83		*N* = 52		*N* = 135^a^	
	n	%	n	%	n	%
Identified as bCPAP-Eligible						
Yes	49	59.0	3	5.8	52	38.5
No	23	27.7	45	86.5	68	50.4
Unknown	11	13.3	4	7.7	15	11.1
bCPAP Initiated						
Yes	43	51.8	2	3.9	36	33.3
No	23	27.7	46	88.5	60	51.3
Unknown	17	20.5	4	7.7	21	17.9

^a^One infant’s eligibility could not be determined

Overall, among the 136 PT/VLBW admitted, 90 (66.2 %) infants survived to discharge, 35 (25.7 %) died and 3 (2.2 %) were referred for tertiary care. Outcome information was missing for 8 (5.9 %) infants. For the 43 infants who were bCPAP-eligible and for whom bCPAP was initiated, 41.9 % (*n* = 18) recovered and 48.8 % (*n* = 21) died (Table 4). Of the 23 bCPAP-eligible infants for whom bCPAP was not initiated, 56.5 % (*n* = 13) recovered, 39.1 % (*n* = 9) died and information about the outcome was missing for 4.4 % (*n* = 1). Outcome information was missing for 1 (2.3 %) infant. A large proportion of infants who were CPAP ineligible recovered whether bCPAP was initiated (100 %, 2 out 2) or not initiated (93.5 %, 43 out of 46). For infants who did receive bCPAP, no complications such as skin injury, pneumothorax or abdominal distention were reported.

**Table 4:** Clinical Outcomes for Preterm (<33 weeks) or very low birth weight infants (<1500 g) with and without bCPAP intervention

	Recovered/ Discharged		Died		Transferred for care		Outcome unknown	
	*N* = 89		*N* = 35		*N* = 3		*N* = 8	
	n	%	n	%	n	%	n	%
Eligible								
bCPAP Initiated (*N* = 43)	18	41.8	21	48.8	3	6.9	1	2.3
bCPAP Not Initiated (*N* = 23)	13	56.5	9	39.1	0	0	1	4.4
bCPAP Initiation Unknown (*N* = 17)	10	58.8	4	23.5	0	0	3	17.7
Not eligible								
bCPAP Initiated (*N* = 2)	2	100	0	0	0	0	0	0
bCPAP Not Initiated (*N* = 46)	43	93.5	0	0	0	0	3	6.5
bCPAP Initiation Unknown (*N*=4)	3	75.0	1	25.0	0	0	0	0

## Discussion

In this study, we assessed the implementation of bCPAP with PT/VLBW infants at three district hospitals in rural Rwanda and found the intervention feasible in a resource-limited rural setting. Over the nine-month period, 45 infants were initiated on bCPAP, demonstrating that bCPAP – an evidence-based intervention to improve survival or PT/VLBW infants – is filling a medical care need for neonates. However, only 52 % of bCPAP-eligible infants received bCPAP, suggesting ongoing gaps in correct identification and initiation of eligible infants. We suspect that this low sensitivity might be a result of turnover of nurses and doctors and could be improved with increased onsite mentorship and refresher trainings, particularly to identify early and mild signs of distress promptly for immediate CPAP initiation to gain the full benefit of the intervention. Qualitative research to assess and understand the barriers to implementation experienced by nurses and doctors is also advised.

Conversely, 88.5 % of bCPAP ineligible infants were not initiated, indicating that clinicians are not exposing ineligible infants to possible bCPAP side effects and conserving the machines for the infants most in need. Only two of the bCPAP initiated infants were bCPAP ineligible according to medical file documentation, an improvement over a study in Malawi where of the 11 neonates treated with bCPAP, six did not meet initiation criteria [16].

A quarter of infants included in this study died before discharge from the hospital. This mortality rate is similar to outcomes of PT/VLBW infants in similar settings in sub-Saharan Africa [18–20]. The highest rate of death in this study, nearly 49 %, occurred in infants eligible for CPAP who died after initiation. Given the low sensitivity of CPAP initiation, we suspect that this group had a higher severity of respiratory distress and other comorbidities compared to infants who were not initiated on CPAP. We were unable to accurately assess the severity of respiratory distress among those who were eligible but not initiated on CPAP; however, we suspect that they were likely to be less severely ill. In addition, our study was conducted in rural hospitals without full-time pediatric specialists on staff; however, similarly high mortality rates among bCPAP initiated infants have been reported in studies conducted in teaching hospitals with more specialized staff [15–17, 21].

There are several limitations to consider for this study. This study is based entirely on routinely collected data available in the patient file. While we cannot verify the accuracy of diagnosis, we believe the information provided by clinicians is reliable because of their clinical background and expertise. For some cases, however, there was limited documentation from clinicians especially on the severity of respiratory distress. Our study excluded term and near-term infants whose bCPAP eligibility depended on the severity of respiratory distress, which was difficult to capture in patients records. Furthermore, we were unable to assess the degree of distress among eligible infants whom were not provided bCPAP to assess for possible selection bias. In a few cases for the PT/VLBW infants, it was difficult to determine whether the infant was identified for bCPAP or initiated on bCPAP. To improve documentation and resulting quality improvement, we recommend the revision of the neonatology patient chart and onsite training/supervision. Despite these challenges, we believe these results are informative as they represent the first assessment of bCPAP implementation in rural Rwanda and thus provide a basis for informing better service delivery and bCPAP scale-up in similar settings.

## Conclusion

To our knowledge, this is the first study of implementation of bCPAP in rural district hospitals in sub-Saharan Africa. We found that bCPAP is a feasible way to support infants with respiratory distress in resource-limited settings. While the introduction and use of bCPAP in this setting appears promising, there remain challenges in terms of guideline adherence. We believe that providing more intense mentorship and refresher trainings can improve guideline adherence, particularly given the high rates of staff turnover. We also recommend the adaption of clinical charts to facilitate clinical determination of degree of respiratory distress and consequent decision-making. Future qualitative and prospective research is needed to determine challenges encountered by clinicians in using bCPAP as well as delineate the reasons for high mortality among infants put on CPAP. Finally and critically, more research is needed to assess the impact of bCPAP on long-term survival and outcomes for PT/VLBW infants.

## Abbreviations

BCPAP: Bubble continuous positive airway pressure
CPAP: Continuous positive airway pressure
GA: Gestation age
ID: Identification
IMB: Inshuti Mu Buzima
MOH: Ministry of Health
PIH: Partners In Health
PT: Preterm
SpO_2_: Oxygen saturation
STROBE: STrengthening the Reporting of OBservational studies in Epidemiology
VLBW: Very low birth weight
